# Neutrophil Extracellular Traps in the Autoimmunity Context

**DOI:** 10.3389/fmed.2021.614829

**Published:** 2021-03-22

**Authors:** Maurizio Bruschi, Gabriella Moroni, Renato Alberto Sinico, Franco Franceschini, Micaela Fredi, Augusto Vaglio, Lorenzo Cavagna, Andrea Petretto, Federico Pratesi, Paola Migliorini, Angelo Manfredi, Giuseppe A. Ramirez, Pasquale Esposito, Simone Negrini, Barbara Trezzi, Giacomo Emmi, Domenico Santoro, Francesco Scolari, Stefano Volpi, Marta Mosca, Angela Tincani, Giovanni Candiano, Marco Prunotto, Enrico Verrina, Andrea Angeletti, Angelo Ravelli, Gian Marco Ghiggeri

**Affiliations:** ^1^Laboratory of Molecular Nephrology, Scientific Institute for Research and Health Care, IRCCS Istituto Giannina Gaslini, Genoa, Italy; ^2^Division of Nephrology and Dialysis Fondazione IRCCS Ca' Granda Ospedale Maggiore, Milan, Italy; ^3^Department of Medicine and Surgery, University of Milan, Bicocca, Italy; ^4^Rheumatology and Clinical Immunology, ASST Spedali Civili and Università of Brescia, Brescia, Italy; ^5^Department of Biomedical, Experimental and Clinical Sciences “Mario Serio”, University of Firenze, and Nephrology and Dialysis Unit, Meyer Children's Hospital, Florence, Italy; ^6^Division of Rheumatology, University and IRCCS Policlinico S. Matteo, Pavia, Italy; ^7^Core Facilities-Proteomics Laboratory, Scientific Institute for Research and Health Care, IRCCS Istituto Giannina Gaslini, Genoa, Italy; ^8^Clinical Immunology Unit, Department of Internal Medicine, University of Pisa, Pisa, Italy; ^9^Unit of Internal Medicine and Immunology, IRCCS Ospedale San Raffaele, Milan, Italy; ^10^Division of Nephrology, University of Genoa and Policlinico San Martino, Genoa, Italy; ^11^Department of Internal Medicine, University of Genoa, Genoa, Italy; ^12^Lupus Clinic Department of Biomedicine, University of Florence, University Hospital Careggi, Florence, Italy; ^13^Nephrology and Dialysis Unit, University of Messina and G. Martino Hospital, Messina, Italy; ^14^Division of Nephrology and Dialysis, University of Brescia and Ospedale di Montichiari, Brescia, Italy; ^15^Division of Paediatric Rheumatology, Scientific Institute for Research and Health Care, IRCCS Istituto Giannina Gaslini, Genoa, Italy; ^16^Rheumatologu Unit, Department of Clinical and Experimental Medicine, University of Pisa, Pisa, Italy; ^17^School of Pharmaceutical Sciences, University of Geneva, Geneva, Switzerland; ^18^Division of Nephrology, Dialysis and Transplantation, Scientific Institute for Research and Health Care, IRCCS Istituto Giannina Gaslini, Genoa, Italy

**Keywords:** *Lupus nephritis*, systemic lupu erythematosus, biomarker, anti-histone, anti-alpha enolase, anti-C1q antibodies

## Abstract

The formation of neutrophil extracellular traps (NETs) is a strategy utilized by neutrophils for capturing infective agents. Extracellular traps consist in a physical net made of DNA and intracellular proteins externalized from neutrophils, where bacteria and viruses are entrapped and killed by proteolysis. A complex series of events contributes to achieving NET formation: signaling from infectious triggers comes first, followed by decondensation of chromatin and extrusion of the nucleosome components (DNA, histones) from the nucleus and, after cell membrane breakdown, outside the cell. NETs are composed of either DNA or nucleosome proteins and hundreds of cytoplasm proteins, a part of which undergo post-translational modification during the steps leading to NETs. There is a thin balance between the production and the removal of circulating NETs from blood where digestion of DNA by circulating DNases 1 and IL3 has a critical role. A delay in NET removal may have consequences for autoimmunity. Recent studies have shown that circulating NET levels are increased in systemic lupus erythematosus (SLE) for a functional block of NET removal mediated by anti-DNase antibodies or, in rare cases, by DNase IL3 mutations. In SLE, the persistence in circulation of NETs signifies elevated concentrations of either free DNA/nucleosome components and oxidized proteins that, in some cases, are recognized as non-self and presented to B-cells by Toll-like receptor 9 (TLR9). In this way, it is activated as an immunologic response, leading to the formation of IgG2 auto-antibody. Monitoring serum NET levels represents a potential new way to herald the development of renal lesions and has clinical implications. Modulating the balance between NET formation and removal is one of the objectives of basic research that are aimed to design new drugs for SLE.

**Clinical Trial Registration Number:** The Zeus study was registered at https://clinicaltrials.gov (study number: NCT02403115).

## Introduction

The pathway(s) leading to the formation of specific autoantibodies to dsDNA in systemic lupus erythematosus (SLE) has long been investigated (1), and mechanisms causing the externalization of DNA and of nucleosome proteins have been a main focus. Exposure to the environment of otherwise hidden molecules such as DNA and nuclear proteins logically represents a possible starting step for autoimmunity.

The formation of neutrophil extracellular traps (NETs) is a special event leading to neutrophil cell death and, as a consequence, to the externalization of DNA and histones. A recent discovery is that a few NET proteins are oxidized by reactive oxygen species (ROS) and undergo a process of post-translational modification by which they are transformed in potential new antigens for autoimmunity (2). The structural characterization of those intracellular proteins that are externalized through NETs and become auto-antigens in SLE has now been completed. The formation of NETs and externalization of NET components are the main foci of research: since DNA and nucleosome are tightly linked in a compacted structure and are not prone to be externalized from the nucleus, solubilization of the nucleosome complex plays a particularly important role.

NET composition and removal are the main topics of the present review that propose NETs as an important source of auto-antigens involved in SLE and, in particular, in lupus nephritis. In this view, NETs would represent an important target for new preventive strategies aimed at blocking the autoimmune process at an early stage, before the generation of autoantibodies.

## Neutrophil Extracellular Traps in Healthy and in Disease

### Pathways for NETs

Neutrophils represent the first line of defense against aggression by bacteria, virus, mumps, and other external potential infectious triggers. One of the strategies that neutrophils utilize for contrasting any external infectious attack is the release of NETs (3, 4). NETosis is a sort of premature cell death that leads to the formation of a physical net where pathogens are entrapped and killed by elastase, defensin, myeloperoxidase, etc. (3, 5, 6). It is a suicidal procedure during which neutrophils die but play their defensive function by also capturing and killing bacteria after their death. Therefore, activation and production of NETs is an important step of innate immunity, and in rare cases, the formation of NETs is reduced for genetic reasons such as NADPH oxidase mutations (see the discussion below) and patients suffering from recurrent and severe infections (7).

The formation of NETs is stimulated by signals that come from outside the cells and then continues with mobilization of granule proteins to the nucleus, decondensation of nuclear chromatin, nuclear membrane dissolution, and then NET externalization. There are several stimuli that can induce the formation of NETs and that can be divided in two major families depending on the participation of infectious or suicidal NETosis or of immunologic triggers, in which cases NETosis is defined as sterile (5, 8). Generation of ROS in the mitochondria has a key role in initiating suicidal NETosis. Ionophores produced by Gram-positive bacteria directly bring calcium inside the mitochondria and induce this process. Specific bacterial toxins such as lipopolysaccharide (LPS) lead to ROS formation *via* an alternative pathway that involves TLR4-dependent NADPH-oxidase activation and suppression of anti-oxidative enzymes (9, 10). ROS generation is, in turn, followed by the activation of several kinases downstream of PKC (i.e., c-Raf, MEK, Akt, ERK) (11–13). Triggers of sterile NETosis include antibodies, cytokines, and inflammation *per se* that activate neutrophil PKC *via* phosphorylation of NADPH-oxidase (14–16).

The second key event is the release of NETs that takes place after activation of neutrophil elastase that dissembles F-actin and moves to the nucleus where it catalyzes the cleavage of histone 1 and de-condenses chromatin; neutrophil elastase also destroys the membrane, allowing DNA to be released outside the cell (17, 18). Enzymatic deimination of arginine residues of histone 1 by peptidylarginine deaminases (especially PAD4) may take place in this phase and play an addictive role in weakening the chromatin backbone (19, 20). Therefore, decondensation and loss of chromatin stability induced by neutrophil elastase, with the contribution of PAD4, is extremely important to modify the rigid structure of chromatin into a fluid compost that is functional for DNA release from the cell.

A less common type of DNA externalization that does not require lysis of neutrophil is “vesicular NETosis,” in which case NETs are released *via* budding from the nucleus and in a vesicular form from the cell. This particular mechanism of NETosis does require modification of cell membrane, maintains neutrophils with vital function, and generates a DNA that is entrapped in micro-vesicles and that requires specific mechanisms of digestion (see below).

### NETs Is a Source of Bio-Available DNA

The physical form of extracellular DNA influences the dynamics and mechanisms of anti-DNA antibody generation, and it is probably critical for DNA removal. In many cases, DNA in NETs or in microvesicles is presented as a DNA–protein complex, including nucleosome proteins, and ideally represents an antigenic source where proteins function as epitopes for B cell presentation (21). In NETs, DNA co-localizes with other non-nucleosome proteins that may, on their own, function as epitopes for auto-antibody formation (22, 23). This part will be discussed below.

The removal of DNA in NET filaments plays a role in antibody generation; since more DNA is digested by DNases, less is the probability that formation of anti-DNA antibodies will be carried out. DNase1 and DNase1L3 are two homologous extracellular enzymes deputed to the removal of circulating bio-available DNA (24). The former enzyme usually digests protein-free DNA; DNases1L3 instead has more powerful functions to digest DNA packed in chromatin and in microvesicles (24–26). Therefore, the bio-availability of extracellular DNA is dependent mainly on the activity of these two enzymes whose importance is strengthened by the finding that genetic conditions carrying molecular defects in *DNASE1 or DNASE1L3* genes are associated with severe forms of pediatric SLE or with other forms of autoimmune disease such as rheumatoid arthritis and sclerodermia (27).

### NET Protein Composition

The literature on the composition and structure of NET proteins is scanty, and only few studies allow the comparison among different auto-immune conditions. The report by Urban et al. (28) is the first complete analysis of NETs deriving from normal neutrophils: these authors utilized western blot and reported a list of 25 proteins that included histones, proteins of granules, cytoplasm, cytoskeleton, and glycolytic enzymes. Petretto et al. (29) analyzed the protein composition of NETs produced spontaneously *ex vivo* by normal neutrophils or after stimuli that mimic an infectious trigger (LPS, calcium ionophores) or sterile triggers (PMA). They found many more proteins in NETs after stimulation of neutrophils and characterized 330 proteins overall. Bruschi et al. (2) analyzed NET proteins produced by neutrophils derived from 33 SLE patients (18 with lupus nephritis) and 21 normal people and reported the presence of NETs in more than 800 proteins overall, mostly belonging to proteins described in the area of autoimmunity, inflammation, and lupus. Many, if not all the proteins found in NETs, presented one or more post-translational modifications (i.e., methionine sulfoxide, thiol oxidation, deamination, phosphorylation) that coexisted in some cases. This is the direct demonstration that post-translational modifications of NET proteins take place *in vivo*. Fifteen proteins maximized the discrimination between SLE and LN (Table 1): four proteins were high in SLE (i.e., VGLL3, MAGOHB, GSTO1, CADPS). The other 11 were instead high in NETs produced by LN patients (GLOD4, MYCBP2, WDR1, ANXA1, ENO1, MPB-ENO1, ESD, NUTF2, DSG1, SYTL3, RAB11FIP1). Two of them, i.e., annexin A1 (ANXA1) and αenolase, were modified for deamination (the former) and for oxidation (the second); αenolase was modified for the presence of methionine sulfoxide in place of methionine 93 (2). Overall, these data give an impressive view of the complex composition of NETs that would be propedeutical to studies on mechanisms.

**Table 1:** List of the fifteen NETs proteins that maximize the discrimination between SLE and LN.

**Protein**	**Gene**	**SLE/LN**	**Modification**
1-Transcription cofactor vestigial-like protein 3	VGLL3	+/−	Nd
2-Protein mago nashi homolog	MAGOHB	+/−	Nd
3-Glyoxalase domain-containing protein 4	GLOD4	−/+	Nd
4-E3 ubiquitin-protein ligase	MYCBP2	−/+	Nd
5-WD repeat-containing protein 1	WDR1	−/+	Nd
6-Annexin A1	ANXA1	−/+	Deamination
7-Alpha-enolase	ENO1	−/+	Oxidation
8-Alpha-enolase MBP-1	ENO1	−/+	Nd
9-S-formylglutathione hydrolase	ESD	−/+	Nd
10-Nuclear transport factor 2	NUTF2	−/+	Nd
11-Glutathione S-transferase omega-1	GSTO1	+/−	Nd
12-Desmoglein-1	DSG1	−/+	Nd
13-Synaptotagmin-like protein 3	SYTL3	−/+	Nd
14-Rab11 family-interacting protein 1	RAB11FIP1	−/+	Nd
15-Calcium-dependent secretion activator 1	CADPS	+/−	Nd

*Four proteins were higher in SLE (VGLL3, MAGOHB, GSTO1, CADPS) the other 11 were higher in LN*.

*Nd, not detected. +/− indicates that the protein is specific for NETs produced by patients with SLE, −/+ means the opposite*.

### NETs in the Immunologic Context

**Figure 1:**
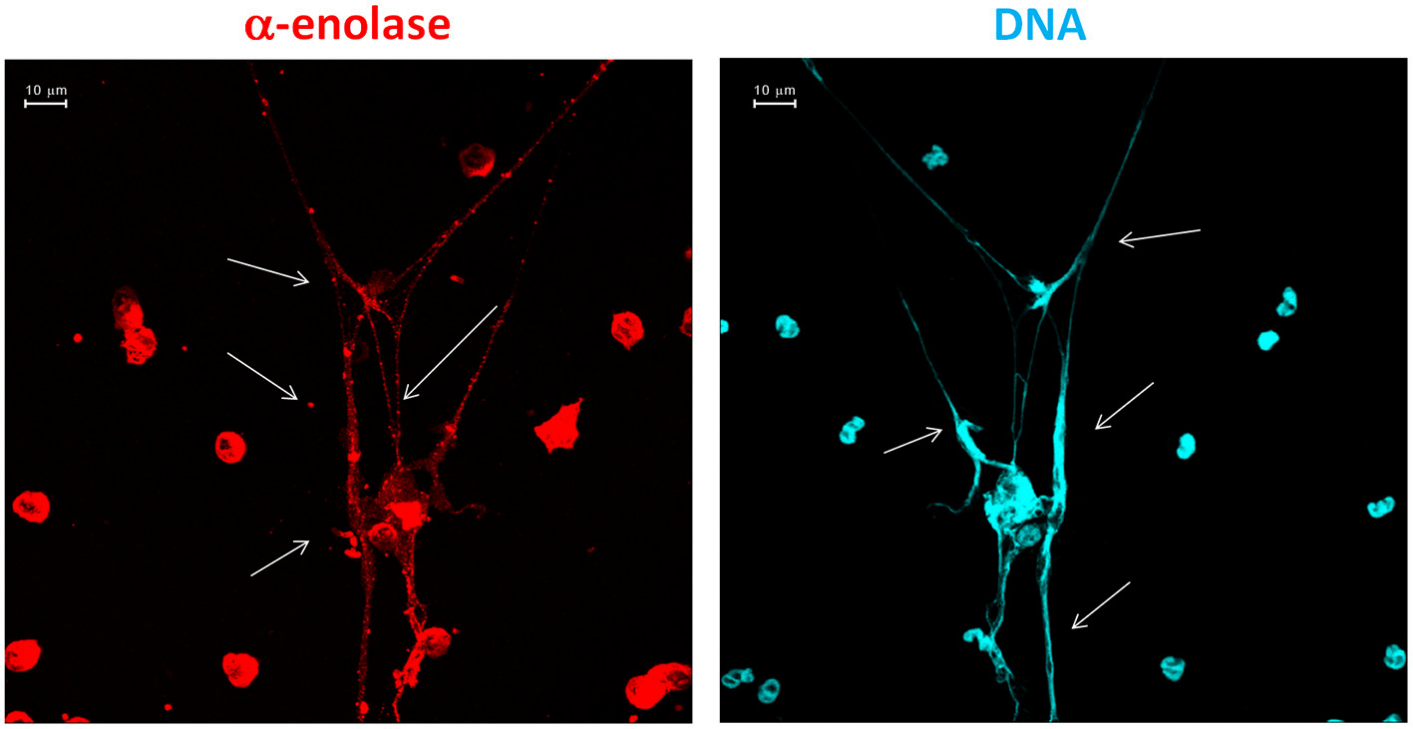
Traditional immunofluorescence microscopy analysis of neutrophil extracellular trap (NET) filaments showing that αenolase and DNA are intensely present in NET filaments and co-localized in large segments. The images were acquired using LSM 510 Meta confocal system scan.

**Figure 2:**
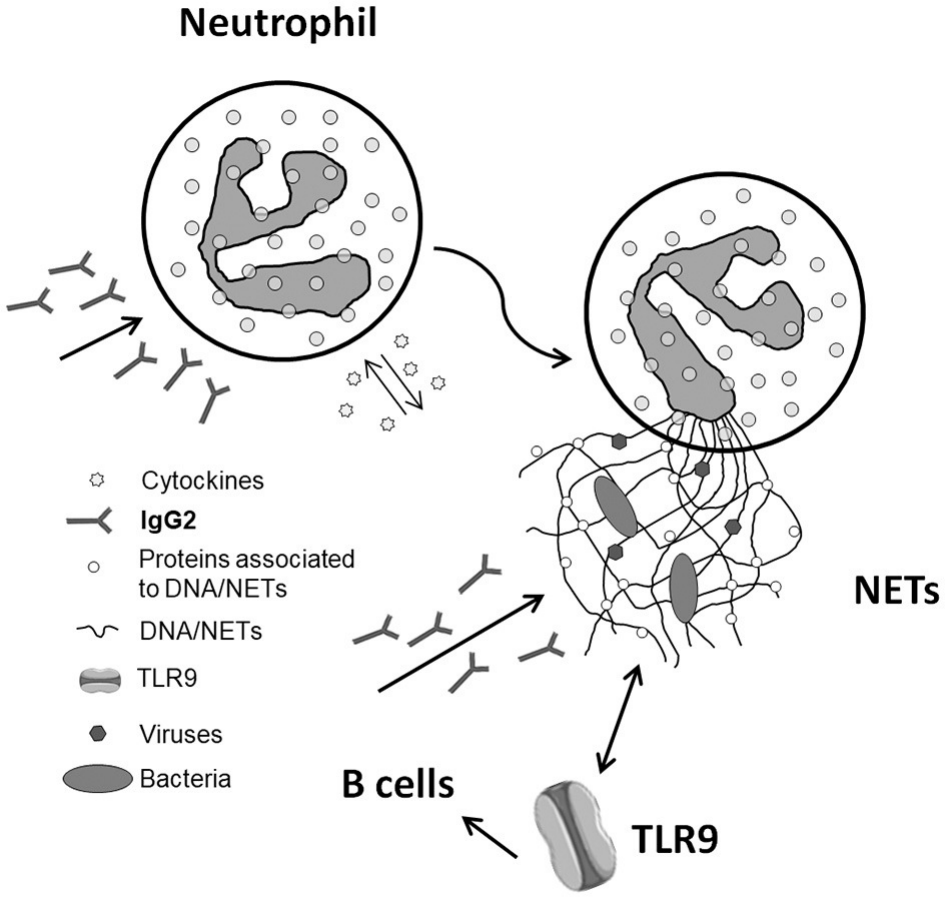
Schematic representation of the pathway that potentially connects neutrophil extracellular traps (NETs) with the formation of antibodies of IgG2 isotype in systemic lupus erythematosus. The various steps are detailed in the text. As for the last step that involves TLR9, the data supporting a direct link between NETs and IgG2 *via* TLR9 are presented in this special issue of *Frontiers* (The kidney in inflammatory and immune-mediated diseases) (see Bertelli et al.).

The implication of NETs in autoimmunity is now a topic of intense discussion (30–32), and SLE is a main focus (33). The interest is about the possibility that NETs are a source of antigens for autoantibodies. A first point concerns DNA since the formation of NETs represents a cell death mechanism according to which DNA is externalized. A second key aspect is the protein composition of NETs and the potential implication of NETs as a source, in addition to DNA, of post-translational modified proteins. One example is αenolase that, in NETs, co-localizes with DNA and is modified by oxidation (Figure 1). Actually, anti-αenolase antibodies of IgG2 isotype represent the major nephritogenic autoantibodies purified in circulation and in the glomeruli of patients with lupus nephritis (22, 23, 34), and studies are now focalizing on the significance of this new class of autoantibodies that identifies particular classes of patients with lupus nephritis (35, 36). There is now consensus in considering IgG2 as the major isotype of autoantibodies in SLE and in LN, and the interest is that IgG2s are secreted upon stimulation of TLR9s that is the class of Toll-like receptors deputed to produce an isotype switching. TLR9s are also the Toll-like receptors that bind DNA. A possibility is that the complex DNA–αenolase in NET filaments is recognized by TLR9s that stimulate B cells to produce anti-DNA and anti-αenolase IgG2 (see the scheme in Figure 2).

### Circulating NET Balance in Healthy and in Disease

Removal of NETs from the circulation is a key event that requires availability of DNases; failure or impairment of this process is a potential starting event leading to autoimmunity. In fact, for the reasons presented above, the persistence of NETs in the circulation would increase the time of exposure of potential antigens to TLR9s and potentiate the autoimmune response (Figure 2).

Circulating NETs can be detected by ELISAs that target the complex of DNA–MPO and/or DNA–elastase that are reported as NET remnants (the second ELISA is more specific for NETs derived from neutrophils and exclude NETs from monocytes); NET remnants are released from the NET complex and are present in free form in serum and biological fluids (37). The evidence that circulating NETs are increased in SLE and in LN and more, in general, in autoimmune conditions has been accumulating in the recent years (38–40). Kessenbrock et al. (41) and Soderberg et al. (42) reported high NET remnant levels, respectively, in serum and plasma of patients with small vessel vasculitis. Knight et al. (43) demonstrate enhanced NET formation in the New Zealand mixed 2328 (NZM) model of murine lupus. Studies in humans confirmed high circulating levels also in SLE and supported their inflammatory potential. Zhang et al. (44) reported high plasma levels of circulating free DNA in patients with SLE that, in part, derived, but not solely, from NETs. Lood showed that SLE individuals had increased plasma levels of myeloperoxidase (MPO)–DNA, and human neutrophil elastase–DNA compared to controls (45). More recently, Bruschi et al. (46) studied 216 patients with SLE and with lupus nephritis, utilizing the assay that immobilizes the MPO tail of the MPO–DNA complex (37). This study showed an increase in circulating NET levels in patients with lupus nephritis and its association with parameters of disease activity such as complement consumption, ESV, RCP, and proteinuria. It is noteworthy that serum NETs were also strongly correlated with anti-dsDNA and anti-C1q antibody levels. On a more clinical vein, determining serum NET remnants represents a potential new biomarker of autoimmunity activity that may occur in concomitance with or may even precede the formation of autoantibodies. The increased level of circulating NETs in lupus nephritis also suggest that the formation of NETs is, in some ways, correlated with the formation of antibody specific for the kidney.

The study by Bruschi et al. (46) also addressed the question of removal of NET remnants by DNASE1 and DNASE1L3 (26). They found normal serum levels of both enzymes in spite of reduced DNase activity; when the same sera were pre-treated with Protein-A in order to remove antibodies, DNase activity was restored. Other authors (40, 47) have already shown the existence, in circulation, of inhibitors of NET removal in patients with SLE. In particular, Hakkim et al. (40) showed that a subset of SLE patients present an impaired degradation of NETs due to the presence of anti-NET antibodies that inhibit DNASE1. These patients, defined as non-degraders, were prone to develop lupus nephritis, suggesting that impaired NET degradation is linked with renal manifestation of lupus. The results of Soderberg et al. (42) reinforce the concept discussed above.

Several data of the literature in humans carrying mutations in DNases (48–51) and results deriving from experimental models (25, 26, 52, 53) indicate a clear association of reduced DNase activity with autoimmune renal lesions. Overall, the data presented above (46) strengthen the concept that circulating inhibitors of DNase activity reduce the removal of NETs and represent a key factor for increasing the exposure of antigens (i.e., DNA and other soluble proteins) to the environment as triggers of autoimmunity.

### NETs–Macrophages Interaction

The interaction of macrophages with NETs is a final aspect that governs the thin balance between a physiological antimicrobial function and autoimmunity. M1 and M2 macrophages act synchronically in this context and are synergic with DNase for removing DNA from NETs. After interacting with NETs, M2 macrophages produce chemotactic substances such as MIF, CCL2/MCP- 1, CCL3/MIP-1a, and CCL4/MIP 1b that recruit monocytes and M1 macrophages that contribute to neutrophil depth and increase, in this way, the quota of extracellular DNA that derives from neutrophils (54). Dead neutrophils are then trapped and cleared by M2 macrophages. In this sense, autoimmunity may derive from an imbalance of the macrophage cycle, resulting in the accumulation of DNA. Therefore, NETs–macrophages interaction is key to the maintenance of the equilibrium between DNA antibacterial functions and autoimmunity and is addictive to DNases to govern the balance between the two. How macrophages and DNases are regulated, whether there is a cross-talk between the two, and which DNA type they process (i.e., chromatin DNA, NET DNA, or microparticle DNA) should be better defined in the future (26).

## Pharmacological Modulation of Net Levels

Modulation of NETs could be functional to reduce the quota of antigens presented to TLRs. Drugs that inhibit NET generation are already available; however, whether or not they offer a real opportunity or play deleterious effects on host defense is not clear and needs new evidence. One possibility is to achieve a correct balance between NET production and removal, and in this sense, modulation of removal after the protective functions of NETs have been obtained could make sense. In the sections below, the possibility to either inhibit formation or enhance removal is briefly outlined.

### Modulation of NET Production With Traditional Drugs

Blocking the first steps of NETosis by means of scavengers of ROS such as N-acetyl-cysteine and/or by inhibitors of NADPH has been already utilized with modest clinical effects in patients with SLE (55). Inhibitors of myeloperoxidase such as 4-aminobenzoic acid hydrazide have been used instead in mouse models of SLE and in vasculitis complicated by glomerulonephritis: the main finding was that they limited the accumulation of neutrophils in the glomeruli and also reduced proteinuria.

One way to reduce NETs is by the inhibition of PAD4. This has been investigated in different animal models, one utilizing mice with constitutional PAD4 deficiency (*PAD4*−*/*−*)* and the other with chemical inhibition of PAD4 by Cl-amidine. In the first case, PAD4-deficient mice were exposed to selected organ pathologies such as pulmonary inflammation, causing acute respiratory distress syndrome. *PAD4*−*/*− mice presented reduced NETosis and a decrease in neutrophil influx into the lung that were accompanied by improved survival compared with wild-type mice (56). In the second case, lupus-prone New Zealand mixed 2328 (NZM) mice, a model of lupus driven by type I IFNs, were treated with Cl-amidine, showing a reduction of NET formation *in vivo* and a significant modification of circulating autoantibody profiles and complement levels followed by reduced glomerular IgG deposition (43).

More recently, we screened a library of biologically active substances utilizing an assay based on high-content imaging and identified vanilloids as a novel class of chemical compounds able to hinder PMA and ionomycin-induced NET release (57). A parallel effect of vanilloids was to decrease cytosolic ROS production, which makes sense in view of the well-known relationship between ROS and NETosis. The identification of a novel class of ROS and NET inhibitors able to stop excessive or aberrant NET production should be considered as an option for treating those disorders associated with NET overproduction.

There are further potential targets for reducing NETosis; a brief list includes the actin cytoskeleton (58), CXCL5, integrins, and TNF (59), all of which may offer interesting therapeutic options.

### Modulation of DNA Removal From NETs

For more than 20 years, inhaled recombinant DNase has been utilized in patients with cystic fibrosis and with other inflammatory lung diseases based on the consolidated finding that this approach plays some positive effects without side effects (60, 61). Treatment with DNase I has also been proposed in neurodegenerative diseases, for example, in patients with dementia in the end-stage of Alzheimer's disease (62), but further and larger interventional studies are required in order to evaluate a positive effect.

It is clear that accelerating the removal of NETs with DNases is an option that would reduce the exposure of bio-available DNA for autoimmunity. Only few studies addressed the potential use of DNases in animal models of SLE and provided contradictory results. In one study (63), recombinant DNase 1 has been found to reduce the generation of autoantibodies and also to improve the outcome of proteinuria and kidney damage in a lupus-prone murine model, but the same findings were not confirmed considering survival as a hard endpoint (64). Recombinant DNase has also been utilized in patients with SLE (65) who tolerated the drug, but clinical results on improving the outcome of the disease are not available and need to be tested in phase II studies. The existence of anti-DNase antibodies in the serum of SLE patients may limit the efficacy and prompt new designs that also consider the combined block of anti-DNase antibodies.

## Conclusions

Data in the literature provide crucial elements that implement the basic findings on NETosis and outline the correlation with autoimmunity (Figure 2). Determining serum NET levels could represent an informative way to herald the development of renal lesions and have a clinical implication. New findings on DNase activity in patients with lupus nephritis also support the idea that NETs accumulate in the serum for a defective removal; an ancillary result is that, in SLE patients, the serum levels of DNaseI and DNaseIL3 are normal, suggesting that DNase inhibition is a potential mechanism for the DNase functional defect. The second main conclusion is that NET composition is highly specific for SLE and lupus nephritis and also includes, beyond DNA/histones, modified proteins (i.e., αenolase with methyl sulfoxide methionine 93 among others). Therefore, NET remnant levels, their kinetics of production and removal, and their composition represent a further advancement with new diagnostic and therapeutic potential implications.

## Author Contributions

GG and AR were the principal investigators of the study and were involved in study design and coordination, patients' recruitment, data managing and supervision, manuscript writing, and discussion. MB had a key role in lab analysis, proteomics, supervision, statistics, and data managing. GC, AP, and MP were involved in lab analysis. GM, FF, MF, AV, LC, FP, PM, MB, AM, GR, PE, SN, LC, BT, GE, GG, DS, FS, SV, MM, and AT were involved in the Zeus study and manuscript discussion. All authors contributed to the article and approved the submitted version.

## Conflict of Interest

The authors declare that the research was conducted in the absence of any commercial or financial relationships that could be construed as a potential conflict of interest.
